# Domain wall motion in Pb(Zr_0.20_Ti_0.80_)O_3_ epitaxial thin films

**DOI:** 10.1038/s41598-017-03757-y

**Published:** 2017-06-13

**Authors:** C. Borderon, A. E. Brunier, K. Nadaud, R. Renoud, M. Alexe, H. W. Gundel

**Affiliations:** 1 IETR UMR CNRS 6164, Université de Nantes, 2 rue de la Houssinière, 44322 Nantes, France; 2 University of Warwick, Department of Physics, Gibbet Hill Road, Coventry, CV4 7AL United Kingdom; 3 GREMAN, CNRS UMR 7347, 16 rue Pierre et Marie Curie, 37071 Tours Cedex 2, France

## Abstract

Two Pb(Zr_0.20_Ti_0.80_)O_3_ samples of different thickness and domain configuration have been studied. The *c*-domain sample was found to have a higher coercive field *E*_*c*_ and higher dielectric losses than the other which presents approximately 60% of *c*-domains and 40% of *a*-domains as observed by piezo force microscopy (PFM) characterization. Hyperbolic law measurements reveal that the higher coercive field is due to domain wall pinning in deeper defects and hence a higher field *E*_*th*_ is required for unpinning. The dissipation factors due to domain wall motion, however, are similar in both samples since the domain wall density is low and there is almost no interaction between domain walls. The higher dielectric losses in the *c*-domain oriented sample are a result of a greater contribution from the lattice and seem to be due to strain from the substrate, which is not relieved in a thin sample. PFM and dielectric characterization are complementary methods which provide a better understanding of the domain wall motion.

## Introduction

The dielectric response of domain wall motions have been already investigated for several years in polycrystalline Pb(Zr,Ti)O_3_ (PZT) thin films^1–4^ which present a great contribution of domain wall jump to the permittivity. In order to study those domain wall movements, the Rayleigh law has been employed at low field (*E* < *E*_*C*_/2) to maintain the domain wall density (Rayleigh region)^5–8^. This law is composed of two coefficients where one corresponds to the domain wall pinning/unpinning (jump) phenomena. Recently, however a hyperbolic law which is more complete has been developed^9^. This law permits the contribution from domain wall vibration and the lattice to be distinguished. The dielectric permittivity is then described as a function of the amplitude of the exciting electric field^9^:

1 $${\varepsilon }_{r}={\varepsilon }_{rl}+{\varepsilon }_{rw}={\varepsilon }_{rl}+\sqrt{{\varepsilon }_{r-rev}^{2}+{({\alpha }_{r}{E}_{0})}^{2}}.$$

This relation is valid for real and imaginary part of the permittivity. *E*_0_ is the amplitude of the electric field *E*(*t*) = *E*_0_ sin *ωt*. *ε*_*rl*_ is the intrinsic lattice contribution which has its origin from the deformation of the unit cell^5, 6^, and *ε*_*r−rev*_ corresponds to domain wall vibration which is a reversible process and can be a measure of the domain wall mobility^5^. The sum of these two parameters corresponds to the initial permittivity (when *E*_0_ = 0). The parameter *α*_*r*_ represents the linear dependence of the permittivity on the exciting electric field. It is associated with domain wall pinning and is related to an irreversible modification of the local polarization^5, 10^. All parameters depend on the crystal structure but *α*_*r*_ is especially sensitive to the presence of impurities, dopants or defects^11^. The parameter *α*_*r*_ is generally high in polycrystalline PZT thin films (~1 × 10^−5^ m/V)^12, 13^ because the jump distance is important due to the material’s columnar structure and the low degree of domain wall pinning. As a consequence, the threshold field *E*_*th*_ necessary for unpinning is low and the Rayleigh region (linear variation of the permittivity) is clearly visible. In the present study we address the contribution of the ferroelastic 90 degree domain walls by studying epitaxial PZT thin films, one is mainly *c*-domain oriented and the other consists of *c* and *a*-domains. These two distinct structures allow us to correlate the polarization mechanisms to the respective domain wall motions.

## Experimental Procedure

The Pb(Zr_0.2_Ti_0.8_)O_3_ (PZT) films were synthesized by pulsed-laser deposition (PLD) onto (001)-oriented SrTiO_3_ (STO) substrates coated with a SrRuO_3_ (SRO) layer which is used as a bottom electrode. The PZT composition was chosen because it has a lattice parameter of 3.935 Å which is close to that of SRO (3.928 Å)^14^. Two (001) oriented samples of thickness of 60 nm and 100 nm have been fabricated using similar process as in ref. 14. Briefly, the films were deposited using KrF excimer laser with an energy fluence of approximately 1 J/cm^2^ on vicinal STO obtained by standard etching and annealing process of nominal 0.1° miscut crystals. Nominal 25 nm thick SRO epitaxial layers were firstly deposited at 700 °C in 150 mbar O_2_ pressure followed by PZT deposited at 600 °C in 250 mbar O_2_ pressure. The thickness of the PZT layer was chosen below and above the critical thickness at which misfit strain relaxes in order to obtain one fully strained PZT film showing only *c*-domains and one partly relaxed film showing mixed *a*/*c*-domains. Detailed microstructural investigations have been done in ref. 14.

**Figure 1:**
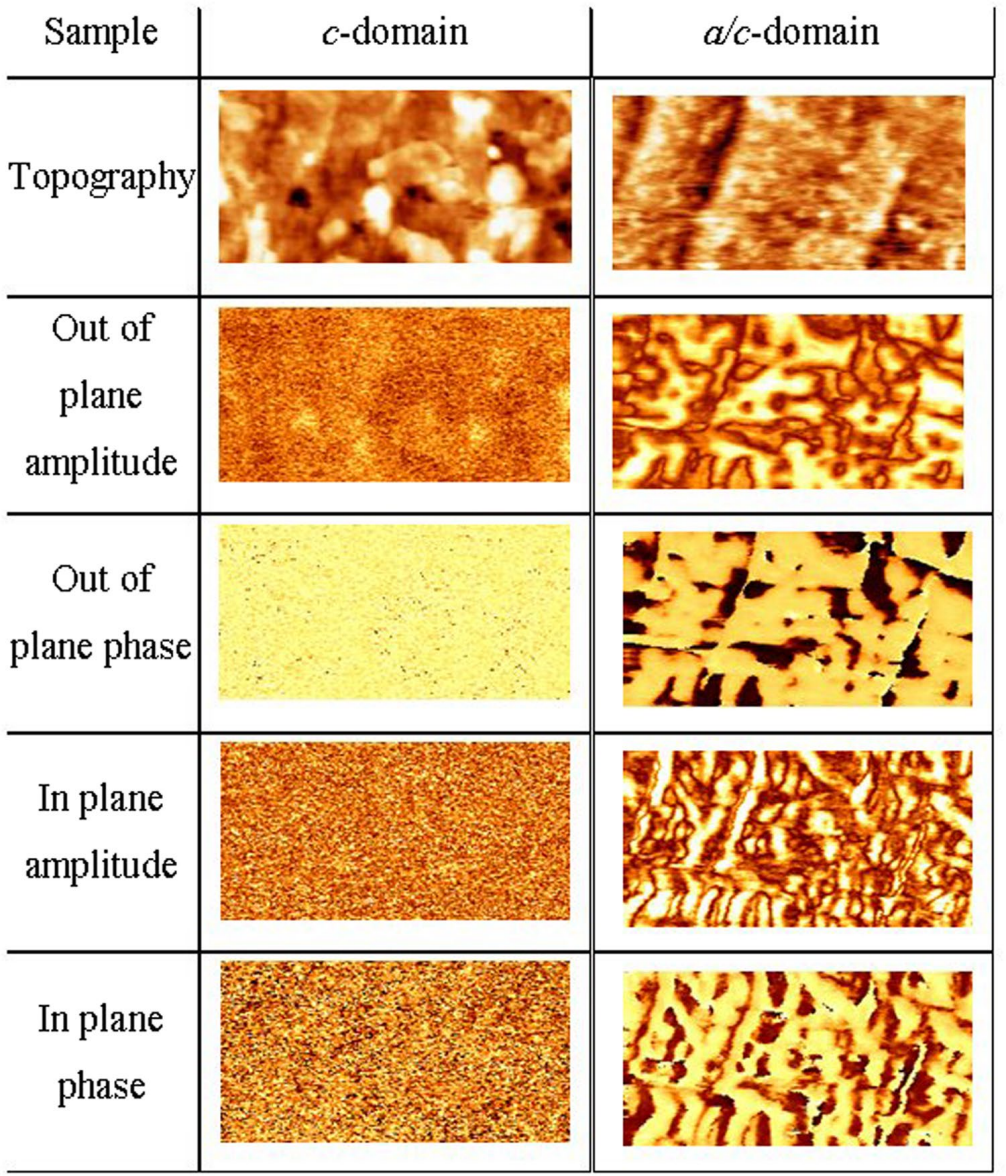
Piezoelectric Force Microscopy (PFM) characterization of the epitaxial PZT thin films. The scan size is 2 μm × 1 μm for the *c*-domain sample and 1 μm × 0.5 μm for the *a*/*c*-domain sample.

The surface morphology of the two samples was investigated by piezoelectric force microscopy (PFM) using a Park XE-100 AFM with ac voltage of 2 V and frequency of 22.36 kHz. ATEC EFM 20 tips were used for the *a*/*c*-domain sample and Pt coated NSC14 tips for the *c* domain sample respectively. Stanford SR830 lock in amplifiers were used and out of plane and in plane PFM images were obtained simultaneously. The amplitude and phase characterization show the two samples (Fig. 1). The phase and amplitude images of the sample of 60 nm thickness have no variation in contrast suggesting that this sample seems to be mainly *c*-oriented. For the sample of 100 nm thickness, the PFM images show different regions and indicate that *a*-domains embedded in a *c*-domain matrix make up approximately 40% of the sample. In a thin layer the strain from the substrate is not relieved by the formation of ferroelastic 90 degree domain walls and the sample is mostly *c*-domain^14^. In the thicker PZT layer the total strain energy is relaxed by the formation of a polydomain structure in the epitaxial films^14^. Thus, we have two samples with similar crystallographic characteristics (both oriented (001)) but with different domain configurations.

Circular platinum electrodes of 160 µm radius were deposited by RF sputtering on both samples in order to realize a Metal-Insulator-Metal (MIM) capacitor. The *P-E* loops have been measured using ferroelectric analyzer (aixaCCt TF2000E) at 300 K and 25 kHz to avoid dielectric losses at low frequencies. The capacitance and the dielectric losses tan *δ* have been determined with an Agilent 4294 A impedance meter at an AC-field amplitude of *E*_0_ = 5 kV/cm. The real and imaginary parts of the permittivity have been calculated from the capacitance and the dielectric loss values. The variation of the permittivity has been measured at 10 kHz as a function the AC field amplitude (0.5 kV/cm to 100 kV/cm corresponding to an applied voltage from 5 mV to 1 V, respectively). All the measurements were performed at room temperature (20 °C).

## Results and Discussion

**Figure 2:**
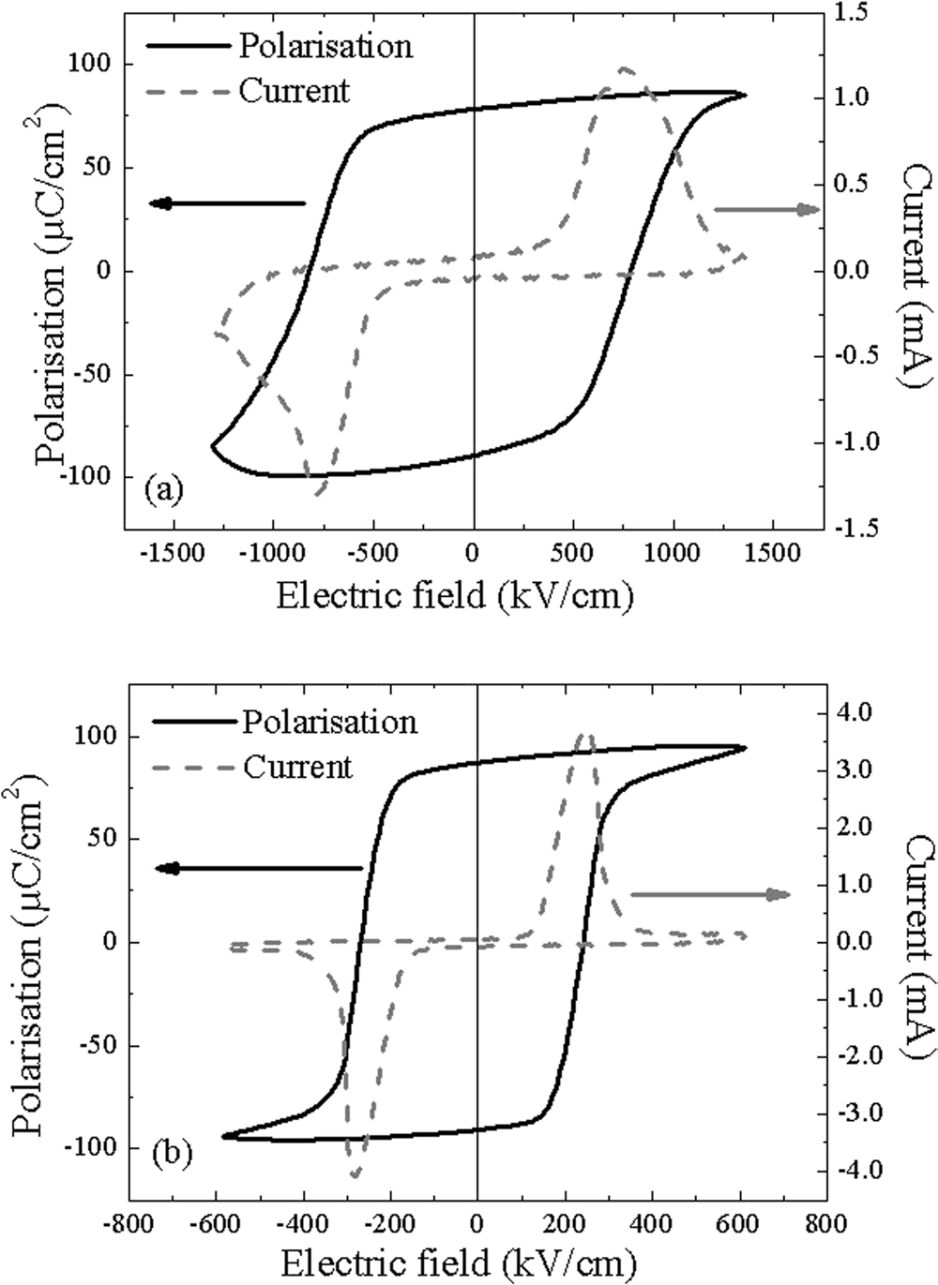
Ferroelectric polarizations and switching current hysteresis loops of (**a**) *c*-domain and (**b**) *a*/*c*-domain samples.

Ferroelectric polarization and switching current hysteresis loops are shown in Fig. 2 for the two samples. The *c*-domain sample has a square hysteresis loop with a high remnant polarization (*P*_*r*_ ≈ 95 ± 5 µC/cm²) which is almost equal to the saturation polarization *P*_*S*_. This value is close to the value reported for PZT epitaxial films of the same composition^15, 16^. The polarization hysteresis curve of the *a*/*c*-domain sample has also a square shape, typical of hard ferroelectric. The remnant polarization *P*_*R*_ is similar to the *c*-domain sample, the coercive field, however, is considerably lower (*E*_*c*_ ≈ 280 ± 10 kV/cm for the *a*/*c*-domain sample whereas *E*_*c*_ ≈ 780 ± 10 kV/cm in the case of the *c*-domain sample). As a consequence, the *c*-domain sample is less saturated than the *a*/*c*-domain sample which is easier to polarize due to the strain relieved with the presence of *a*-domains. The polydomain sample hence can be more easily polarized. The c-domain sample presents also a larger distribution of coercive field which indicates the presence of a higher dc conductivity of fully strained high quality PZT films. This high conductivity is due to a higher mobility related to a low density of extended defects (mainly threading dislocations) and to an influence of polarization on the Schottky barrier. Both effects lead to a higher conductance as a direct influence on the Schottky–Simmons conduction mechanism, as shown in previous work on similar films^17^.

**Figure 3:**
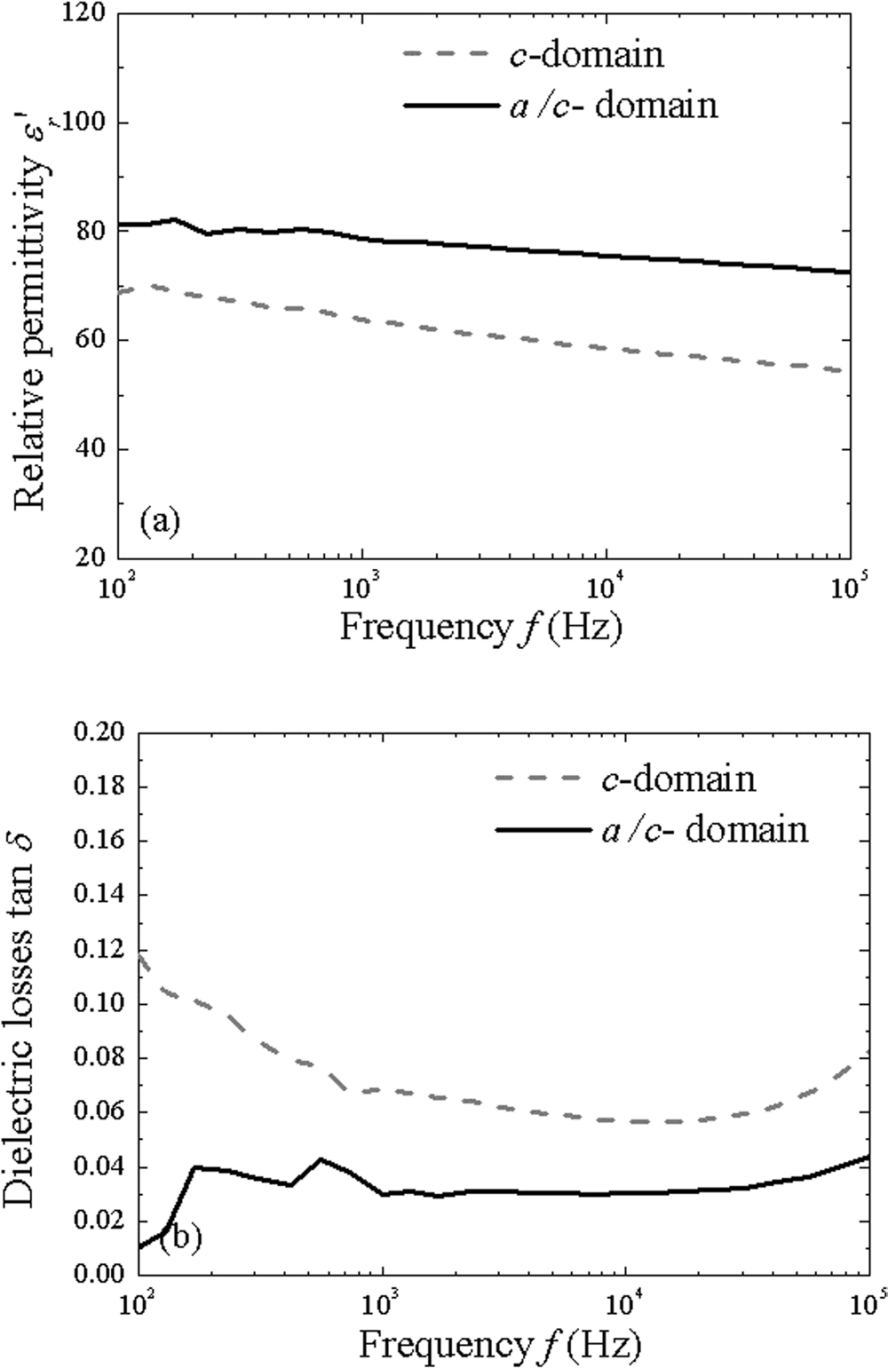
Dielectric properties (**a**) ${\varepsilon }_{r}^{^{\prime} }$ and (**b**) tan*δ* as a function of frequency (*E*_0_ = 5 kV/cm) for the two samples.

The dielectric constant ${\varepsilon }_{r}^{^{\prime} }$ and losses tan *δ* were measured as a function of frequency (Fig. 3). The permittivity of the *c*-domain sample is smaller (${\varepsilon }_{r}^{^{\prime} }$ ~ 58 at 10 kHz) but of the same order than this of the *c* and *a*-domains (${\varepsilon }_{r}^{^{\prime} }$ ~ 75 at 10 kHz). In ferroelectric materials, the permittivity of a polydomain structure is greater than that of an oriented one^14, 18^. This can be seen, for example, from the *C-V* curve where the permittivity is higher for a field near the coercive field due to the domain walls interaction^18^. The dielectric permittivity in the *c*-domain sample is the intrinsic value of dielectric permittivity, as there is no domain wall which may extrinsically enhance it ref. 19. The dielectric losses observed in the *a*/*c*-domain sample are lower than in the *c*-domain sample. This does not correspond to what has been reported before where the polydomain structure exhibits greater dielectric losses due to domain wall interactions^18^. The higher dielectric losses in our case may be due to the strain from the substrate which is not (or less) relieved in the case of the *c*-domain sample.

**Figure 4:**
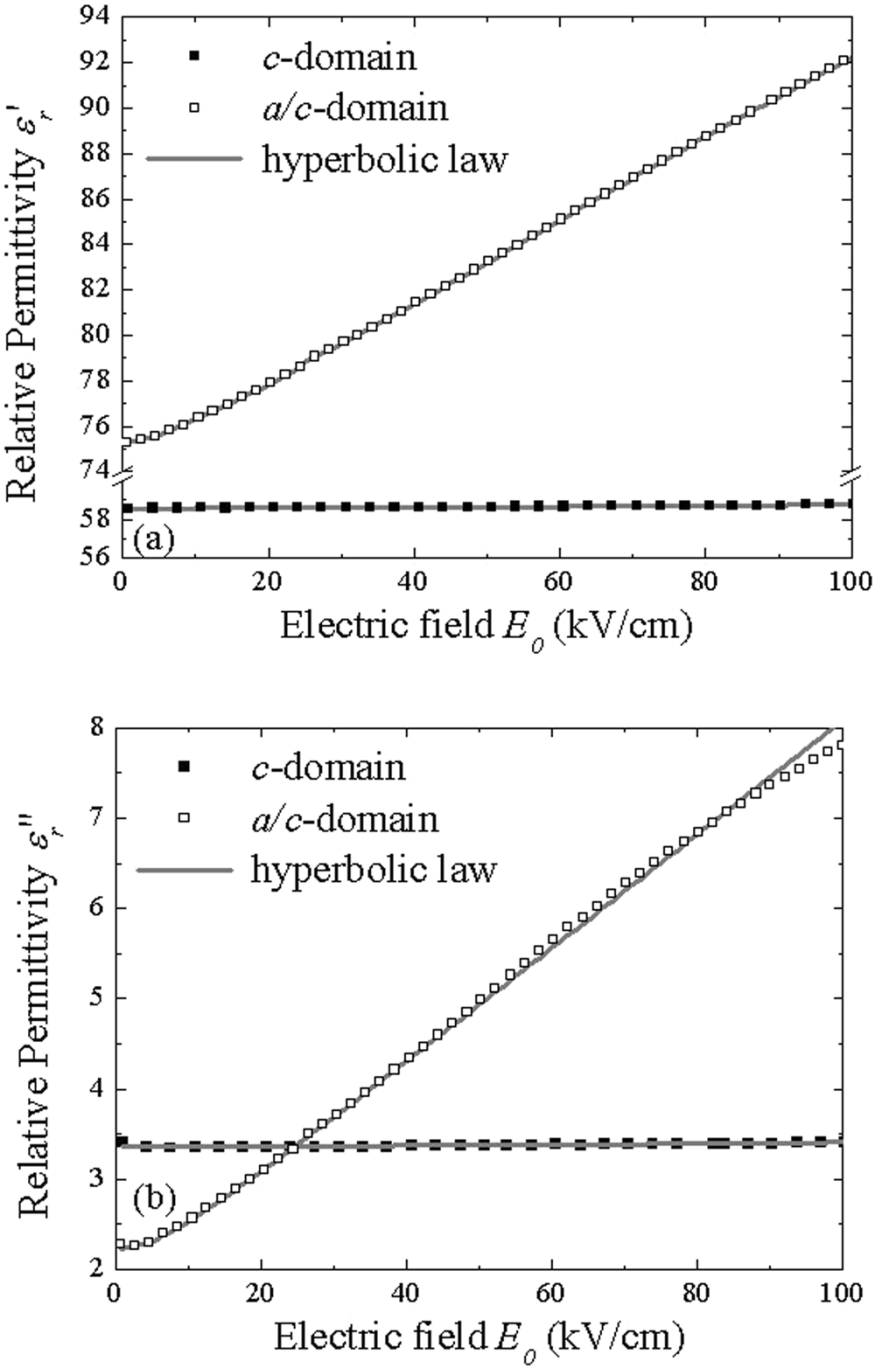
(**a**) Real and (**b**) imaginary part of the permittivity as a function of AC field amplitude (f = 10 kHz) for the two samples.

In order to get a better understanding of the mechanisms responsible of the higher dielectric losses in the *c*-domain sample, the hyperbolic law has been used for decomposing the different components of the real and imaginary parts of the permittivity. The two parts of the permittivity (Fig. 4) can be perfectly fitted with the hyperbolic law (equation (1)). The evolution of the *c*-domain sample is less marked which shows that this sample has a lower contribution of domain wall pinning/unpinning. The calculated coefficients of the respective contributions are shown in Table 1. For the real part of the permittivity, the lattice parameter ${\varepsilon }_{rl}^{^{\prime} }$ is higher in the case of the *a*/*c*-domain sample due to its polydomain structure. The parameter ${\varepsilon }_{r-rev}^{^{\prime} }$ due to reversible domain wall vibration is more than ten times larger in this sample than in the *c*-domain sample. According to Boser^20^, this parameter is proportional to the domain wall density. In the *a*/*c-*domain sample, many domains have been observed by the PFM characterizations (Fig. 1) and the dielectric measurements and the hyperbolic law confirms the higher domain wall density. For the pinning/unpinning parameter ${\alpha }_{r}^{^{\prime} }$, the difference between the two samples is still more marked. ${\alpha }_{r}^{^{\prime} }$ in the *c* and *a*-domains sample is seventy times larger than in the *c*-domain sample. Again, according to Boser^19^, this parameter is proportional to ${\varepsilon }_{r-rev}^{^{\prime} }$ and can be expressed as:

2 $${\alpha }_{r}^{^{\prime} }\propto \frac{{f}_{0}{P}_{s}}{\sqrt{N{F}_{1}}}{\varepsilon }_{r-rev}^{^{\prime} },$$

where *f*_0_ is a geometrical factor, *P*_*s*_ is the polarization at saturation, *N* is the number of obstacles or defects and *F*_1_ is a function which is proportional to the pinning depth of the defect. As the two samples are strongly oriented, the polarization will have the same direction and the factor *f*_0_ *P*_*s*_ will be very similar. This can be seen from the *P-E* loops (Fig. 2) where the remnant polarization is almost identical for both samples. The difference of the parameter ${\alpha }_{r}^{^{\prime} }$ hence will be due to the product *NF*_1._ This indicates that the *a*/*c-*domain sample, which has a higher parameter ${\alpha }_{r}^{^{\prime} }$, has a lower defect density and/or less deep defects. In the *c*-domain sample, defects are more important as the strain is not relieved due to the lower thickness. Moreover, the few domain walls present in this sample are blocked in deep defects. This can be seen from the threshold field for domain wall pinning/unpinning which is defined by ref. 9:

3 $${E}_{th}=\frac{{\varepsilon }_{r-rev}^{^{\prime} }}{{\alpha }_{r}^{^{\prime} }},$$

and is five times higher in the case of the *c*-domain sample. When the threshold field is low much domain walls pinning/unpinning occurs. This means that the domain walls are more free to move in the material. In the case of the *c*-domain sample a very high threshold field is observed (*E*_*th*_ ~ 42.3 kV/cm) and the domain walls are hence blocked. Switching the polarization becomes more difficult and consequently the coercive field is higher. The more important value of the threshold field in the *c*-domain sample is in agreement with the higher value of the coercive field observed in Fig. 2b.

**Table 1:** Coefficient of the lattice, domain wall pinning and domain vibration contributions for the real and the imaginary part of the total permittivity and the dissipation factors of the different contributions.

	${{\boldsymbol{\varepsilon }}}_{{\boldsymbol{rl}}}^{{\boldsymbol{^{\prime} }}}$	${{\boldsymbol{\alpha }}}_{{\boldsymbol{r}}}^{{\boldsymbol{^{\prime} }}}$ ***(µm/V)***	${{\boldsymbol{\varepsilon }}}_{{\boldsymbol{r}}-{\boldsymbol{rev}}}^{{\boldsymbol{^{\prime} }}}$	${{\boldsymbol{\varepsilon }}}_{{\boldsymbol{rl}}}^{^{\prime\prime} }$	${{\boldsymbol{\alpha }}}_{{\boldsymbol{r}}}^{{\boldsymbol{^{\prime\prime} }}}$ ***(µm/V)***	${{\boldsymbol{\varepsilon }}}_{{\boldsymbol{r}}-{\boldsymbol{rev}}}^{^{\prime\prime} }$	***m***_***εrl***_	***m***_***α***_	***m***_***rev***_
*c*-domain sample	58.5	0.026	0.11	3.32	0.008	0.04	0.056	0.31	0.35
*a/c*-domain sample	73.9	1.832	1.49	1.72	0.635	0.51	0.023	0.35	0.34

Concerning the imaginary part of the domain wall motion coefficients (*ε*_*r*−*rev*_ and *α*_*r*_), the ratio between the two samples is quasi-identical. ${\varepsilon }_{r-rev}^{^{\prime\prime} }$ and ${\alpha }_{r}^{^{\prime\prime} }$ are more than ten times higher in the *a*/*c*-domain sample. Consequently, the dissipation factors of the domain wall vibration *m*_*rev*_ and of the pinning/unpinning *m*_*α*_ of the two samples are of the same order. These factors correspond to the ratio between the imaginary and the real parts of the respective contribution and are reported in Table 1. The *m*_*rev*_ dissipation factors are equal to 0.34 and 0.35 and are similar to those that reported in the literature for BST thin films^11, 18^, indicating that the vibration of domain walls is a very dissipative phenomenon^11^. No information on the *m*_*rev*_ values for PZT has been found in the literature. The dissipation parameters *m*_*α*_ are lower than those reported for PZT thin films (*m*_*α*_ ≈ 0.42)^10, 21, 22^. This might be due to the lower domain wall density in the two samples studied, as a higher density favors domain wall interaction and hence a higher dissipation factor^18^. The similar dissipation factor retrieved by our study indicates that the dissipation due to domain wall motion is not sensible to the domain wall density. According to Boser^20^, the ratio between the coercive field and the threshold field is equal to:

4 $$\frac{{E}_{C}}{{E}_{th}}=\frac{2}{\sqrt{\pi }}\sqrt{\mathrm{ln}\,\frac{{L}_{3}}{2{L}_{0}},}$$

where *L*_3_ is the distance between two domain walls and *L*_0_ is the distance between two zero positions of the function which describes the distance between two defects. In our study the ratio is equal to 18 and 34 for the *c*-domain sample and the *a*/*c*-domain sample respectively, which is high compared to what is obtained by Boser (*E*_*C*_/*E*_*th*_ ≈ 2.2)^20^. The distance between domain walls is apparently so large that there is no interaction between them and the domain walls motions are not influenced by the domain wall density. As a consequence, the dissipation factors are identical for the two samples.

The dissipation of the lattice parameters ${m}_{\varepsilon rl}$ is two times higher in the *c*-domain sample than in the *a*/*c*-domain sample. This means that the higher dielectric losses in the *c*-domain sample is due to its structure, probably caused by the strain which is not relieved in the thinner film. Consequently, the *a*/*c*-domain sample has lower global dielectric losses.

## Conclusion

Two (001) oriented PZT samples of a different thickness have been studied. PFM characterization shows a majority of *c*-domains for the 60 nm thick sample whereas the sample of 100 nm thickness presents approximately 60% *c*-domains and 40% *a*-domains. The higher domain wall density in the *c* and *a*-domain sample is confirmed by the hyperbolic law measurements which show that the coefficients ${\alpha }_{r}^{^{\prime} }$ and ${\varepsilon }_{r-rev}^{^{\prime} }$ describing domain wall motion are higher. In the case of the *c*-domain sample the threshold field *E*_*th*_ is higher suggesting that the domain walls needs a higher energy to be unpinned. The mobility of the domain walls in this sample is reduced and a higher coercive field on the *P-E* loop is then observed. This sample presents also higher dielectric losses but the dissipation factors due to domain wall motions are similar to those of the *c* and *a*-domain sample. The higher dielectric losses seem to be due to the strain which increases the lattice losses. The combination of PFM and dielectric characterization in the present work has provided a more complete understanding of the dynamical behavior of domain wall motion in this system than has been previously possible. This study presents one aspect of the switching mechanism, which is the domain wall mobility under high field. Understanding this domain walls mobility allow us to better understand and potentially quantify the field dependence of ferroelectric switching.
